# HbA1c recording in patients following a first diagnosis of serious mental illness: the South London and Maudsley Biomedical Research Centre case register

**DOI:** 10.1136/bmjopen-2022-069635

**Published:** 2023-07-18

**Authors:** Nikeysha Bell, Gayan Perera, David Chandran, Brendon Stubbs, Fiona Gaughran, Robert Stewart

**Affiliations:** 1 Psychological Medicine, King’s College London Institute of Psychiatry, Psychology and Neuroscience, London, UK; 2 Institute of Psychiatry, Psychology and Neuroscience, Psychosis Studies, King’s College London, London, UK; 3 National Psychosis Service, South London and Maudsley NHS Foundation Trust, London, UK

**Keywords:** Health informatics, General diabetes, EPIDEMIOLOGY, Quality in health care, MENTAL HEALTH, PSYCHIATRY

## Abstract

**Objectives:**

To investigate factors associated with the recording of glycated haemoglobin (HbA1c) in people with first diagnoses of serious mental illness (SMI) in a large mental healthcare provider, and factors associated with HbA1c levels, when recorded. To our knowledge this is the first such investigation, although attention to dysglycaemia in SMI is an increasing priority in mental healthcare.

**Design:**

The study was primarily descriptive in nature, seeking to ascertain the frequency of HbA1c recording in the mental healthcare sector for people following first SMI diagnosis.

**Settings:**

A large mental healthcare provider, the South London and Maudsley National Health Service Trust.

**Participants:**

Using electronic mental health records data, we ascertained patients with first SMI diagnoses (schizophrenia, schizoaffective disorder, bipolar disorder) from 2008 to 2018.

**Outcome measures:**

Recording or not of HbA1c level was ascertained from routine local laboratory data and supplemented by a natural language processing (NLP) algorithm for extracting recorded values in text fields (precision 0.89%, recall 0.93%). Age, gender, ethnic group, year of diagnosis, and SMI diagnosis were investigated as covariates in relation to recording or not of HbA1c and first recorded levels.

**Results:**

Of 21 462 patients in the sample (6546 bipolar disorder; 14 916 schizophrenia or schizoaffective disorder; mean age 38.8 years, 49% female), 4106 (19.1%) had at least one HbA1c result recorded from laboratory data, increasing to 6901 (32.2%) following NLP. HbA1c recording was independently more likely in non-white ethnic groups (black compared with white: OR 2.45, 95% CI 2.29 to 2.62), and was negatively associated with age (OR per year increase 0.93, 0.92–0.95), female gender (0.83, 0.78–0.88) and bipolar disorder (0.49, 0.45–0.52).

**Conclusions:**

Over a 10-year period, relatively low level of recording of HbA1c was observed, although this has increased over time and ascertainment was increased with text extraction. It remains important to improve the routine monitoring of dysglycaemia in these at-risk disorders.

**Strengths and limitations of this study**

- We believe this is the first study of this kind.
- Seeking to understand whether HbA1c is being recorded/sufficiently recorded in the serious mental illness population (a current National Health Service target).
- High precision and recall for the natural language processing algorithm (precision 0.89%, recall 0.93%).
- Focus on specific catchment area.
- Does not account for information within primary care.

## Background

People with serious mental illness (SMI) have a substantially reduced life expectancy^1^ presenting a major public health challenge. Among a range of specific health inequalities, type 2 diabetes mellitus (T2DM) incidence rates are higher than expected,^2^ potentially accounting for at least some of the mortality gap and providing an important opportunity for health improvement initiatives. As well as unfavourable lifestyle factors, antipsychotic medications may play at least some part in the high prevalence of T2DM through accelerating glucose dysregulation.^3^ Other factors such as diagnostic overshadowing and underscreening and subsequent treatment could also compound this issue and have been demonstrated in people with established SMI with cardiovascular disease.^4^ Identifying predictors of emergent glucose dysregulation would allow interventions to be directed towards those most at risk, thus creating potential for more precise approaches to clinical and preventative care to reduce the risk of diabetes. In order to have maximum effectiveness, early screening is essential.

Despite these opportunities, further work is needed to clarify how feasible it is to predict T2DM, identify it earlier, or mitigate against its complications in people affected by SMI. Glycated haemoglobin (HbA1c) is a commonly used measure of dysglycaemia and is expected to be regularly recorded in people receiving mental healthcare; for example, it is recommended for English mental health services.^5^ Investigating HbA1c recording levels in routine electronic health records (EHRs) is an important first step in this process, including whether this is consistent across all levels of treatment and care; however, to our knowledge this has not received previous attention. First diagnosis in people with psychotic disorders has been identified as the optimal time to screen and prevent physical health conditions developing, including T2DM, but limited data are available on screening levels in this critical time period.^6^

The aim of this study was to investigate rates of HbA1c recording in the electronic mental health records of people with newly diagnosed SMI (schizophrenia, schizoaffective disorder or bipolar disorder presenting for the first time) receiving care from a large mental healthcare provider. Further, we specifically sought to investigate the factors associated with HbA1c levels being recorded or not (including changes in recording rates over time), to determine the extent to which ascertained HbA1c recording could be increased through a novel natural language processing (NLP) algorithm applied to free text, and to describe distributions of HbA1c levels when recorded.

## Methods

### Sample

The analysed sample was assembled using the Clinical Record Interactive Search (CRIS) data resource at the South London and Maudsley National Health Service (NHS) Foundation Trust (SLAM). SLAM is one of Europe’s largest mental healthcare providers, serving over 1.2 million residents of four south London boroughs (Lambeth, Southwark, Lewisham, Croydon). With services covering all ages and specialties, SLAM has fully used EHRs for over 10 years and its CRIS platform, set up in 2008,^7^ allows researcher access to de-identified data from the full record within a robust governance framework. Because large volumes of information in mental health clinical records are primarily recorded in text, a range of constructs are now quantifiable at scale using NLP algorithms developed for CRIS.^8^

Using CRIS, we extracted data on all patients with a first diagnosis of contact between 1 January 2008 and 31 December 2018 that resulted in a recorded diagnosis of schizophrenia, schizoaffective disorder or bipolar disorder. As described in the Results, analysed data were subsequently restricted to those from 2010 onwards because of limited data for earlier years. If the patient had more than one HbA1c result, the result closest to diagnosis date was used.

### Measures

Serum HbA1c data were available from two sources: (1) Prestructured imported data from local laboratories from which this assay had been requested by SLAM; (2) Information recorded in free-text fields such as case notes and clinical correspondence. In order to render information available for analysis from the latter, a rules-based NLP algorithm was created to identify HbA1c as a recorded entity and to extract the numerical value attributed to that assay. From a corpus of 250 randomly selected documents, annotation features were first built over a training set. The algorithm rules sought to identify instances of HbA1c recording and the associated score where this was being recorded in relation to the patient (ie, rather than anyone else). Any units (% or mmol/mol) were allowable and any number of decimal places (if any). Primary exclusions were HbA1c mentions without a score—for example, text simply indicating that levels were within the normal range or that a measurement had taken place (ie, without a value described). The algorithm derived from this training set was then evaluated against manually annotated gold standards. Once the precision (positive predictive value) and recall (sensitivity) were judged to be at a good enough standard in the development phase, the algorithm was run over CRIS as a whole using a cloud-based processing platform and the results compiled and evaluated formally. The developed NLP algorithm achieved a precision of 89% and a recall of 93% against the gold standard in an independent manually annotated evaluation data set extracted from CRIS.

The following covariates were additionally extracted: age, gender, ethnicity, first SMI diagnosis recorded (categorised into schizophreniform (including schizophrenia or schizoaffective disorder) or bipolar), and the year of first recorded diagnosis. Recorded glucose level was further evaluated as a measure of dysglycaemia.

### Statistical analysis

The sample was initially described and unadjusted associations were done between first SMI diagnosis with HbA1c recording or not as a binary outcome, first investigating laboratory data alone and then this was supplemented with data from the NLP algorithm. Next, a logistic regression analysis was used to investigate independence of these associations, entering all covariates simultaneously. Finally, given low levels of recording in earlier years, a sensitivity analysis was carried out restricting the sample to those diagnosed in 2010 or after.

### Patient and public involvement

At project planning stage, members of a Service User Advisory Group were shown initial ideas and plans and asked for thoughts and suggestions relating to a broader body of work (a PhD programme for NB) of which this study was a component. The group consisted of services users (of SLAM services) with lived experience of mental health problems. They were very keen on finding out the results of the HbA1c recording and encouraged data linkage to primary care (which was subsequently carried out for a nested cohort of people with pre-existing diabetes, to be published separately). Suggestions were taken on board and informed, in part, some of the research questions.

## Results

A sample of 21 462 patients was initially characterised based on the study inclusion criteria for the years 2008–2018. From the laboratory data, we ascertained 4106 patients (19.1%) with at least one HbA1c result recorded. When we supplemented this with the NLP algorithm, the number with at least one HbA1c result recorded rose to 6901 (32.2%; a 68% increase); however, the further addition of glucose data did not substantially increase recording results further (table 1). The mean (SD) interval between the date of diagnosis and that of the first recorded HbA1c result was 2.1 (2.5) years.

**Table 1:** Sample characteristics and prevalence of data on HbA1c and/or glucose levels

	Number	HbA1c—lab value		HbA1c—lab value or NLP-derived		HbA1c or glucose	
		% present	χ^2^ (df), P value	% present	χ^2^ (df), P value	% present	χ^2^ (df), P value
Total sample	21 462	19.1		32.2		35.2	
Age group, years							
<20	2660	20.0		30.3		35.9	
20–29	5237	26.0	314.9 (4)	37.9	129.9 (4)	40.6	143.36 (4)
30–39	4791	20.1	<0.001	32.4	<0.001	36.0	<0.001
40–49	3712	16.2		30.9		33.2	
50+	5062	12.8		27.8		29.7	
Gender							
Female	10 516	17.9	20.5 (1)	30.1	40.8 (1)	33.2	32.5 (1)
Male	10 942	20.3	<0.001	34.1	<0.001	36.9	<0.001
Ethnicity							
White	10 923	15.0		26.8		30.2	
Black	5138	29.5	467.0 (4)	47.3	663.8 (4)	49.8	584.4 (4)
Asian	1250	22.5	<0.001	36.0	<0.001	38.7	<0.001
Mixed	631	20.3		33.4		36.9	
Other	1748	19.6		32.4		35.2	
Diagnosis year							
2008	1853	11.4		23.0		24.8	
2009	1859	9.8		22.9		24.4	
2010	2067	11.2	730.3 (10)	23.7	458.5 (10)	25.2	588.0 (10)
2011	1985	12.5	<0.001	25.3	<0.001	27.9	<0.001
2012	1961	14.3		27.5		33.0	
2013	2000	18.9		32.3		37.1	
2014	2010	23.4		39.7		43.3	
2015	1990	24.4		38.4		41.1	
2016	2056	29.3		42.0		45.1	
2017	2129	29.7		41.3		43.8	
2018	1552	24.8		36.5		39.7	
Diagnosis							
Schizophrenia/schizoaffective disorder	14 916	21.7	213.2 (1)	36.6	452.1 (1)	39.5	414.7 (1)
Bipolar disorder	6546	13.2	<0.001	21.9	<0.001	25.1	<0.001

NLP, natural language processing.

Factors associated with the recording of HbA1c are displayed in table 1. Considering HbA1c derived from laboratory values or NLP, data were most likely in the 20–29 years age group, in men compared with women, and recording was more common in all minority ethnic groups compared with the white group, particularly so for the black group, and in those with a schizophreniform rather than bipolar disorder diagnosis. Prevalence of recorded HbA1c rose consistently from 2010 apart from the 2018 year of data extraction. In adjusted analyses (table 2), associations with all covariates remained statistically significant; however, ORs were reduced in strength (towards the null) for gender, diagnosis and named ethnic minority groups; they were strengthened for diagnosis year. In those with HbA1c data, mean levels are displayed by covariate status in table 3.

**Table 2:** Logistic regression analysis of factors associated with recorded HbA1c presence in the electronic mental health record for years 2008–2018

Characteristic	Unadjusted model 2008–2018	Mutually adjusted model 2008–2018	Mutually adjusted model 2010–2018
	OR	OR	OR
	(95% CI)	(95% CI)	(95% CI)
Age (10-year increment)	0.93	0.94	0.93
	(0.92 to 0.95)	(0.93 to 0.96)	(0.92 to 0.95)
Female gender	0.83	0.91	0.87
	(0.78 to 0.88)	(0.84 to 0.97)	(0.81 to 0.93)
Ethnicity White	Ref.	Ref.	Ref.
Black	2.45	2.2	2.16
	(2.29 to 2.62)	(2.04 to 2.36)	(2.00 to 2.34)
Asian	1.54	1.43	1.52
	(1.36 to 1.74)	(1.26 to 1.62)	(1.33 to 1.75)
Mixed	1.37	1.21	1.14
	(1.16 to 1.63)	(1.02 to 1.45)	(0.94 to 1.37)
Other	1.31	1.19	1.23
	(1.17 to 1.46)	(1.06 to 1.33)	1.10 to 1.39)
Bipolar disorder diagnosis	0.49	0.57	0.57
	(0.45 to 0.52)	(0.53 to 0.61)	(0.53 to 0.62)
Diagnosis year (per 2008–2018 increment)	1.1	1.13	1.14
	(1.10 to 1.12)	(1.12 to 1.15)	(1.13 to 1.16)

**Table 3:** Distributions of first recorded HbA1c levels

	Number with at least one HbA1c recording	Mean (SD) HbA1c first recorded
Total sample	6901	38.3 (10.5)
Age group, years		
<20	807	35.8 (6.0)
20–29	1985	36.2 (7.2)
30–39	1554	38.1 (9.8)
40–49	1147	40.2 (10.0)
50+	1408	43.5 (14.9)
Gender		
Female	3162	38.0 (9.1)
Male	3736	38.6 (10.8)
Missing		34.0 (2.8)
Ethnicity		
White	2924	37.6 (9.6)
Black	2428	39.1 (11.2)
Asian	450	39.5 (10.1)
Mixed	211	38.1 (9.3)
Other	566	37.6 (7.7)
Missing	322	37.5 (7.2)
Year of referral		
2008	426	39.8 (11.2)
2009	426	40.0 (12.6)
2010	490	39.7 (11.7)
2011	503	39.5 (13.0)
2012	539	38.0 (10.4)
2013	645	38.0 (10.5)
2014	798	37.2 (9.0)
2015	765	38.0 (9.8)
2016	863	38.3 (8.9)
2017	880	37.5 (9.5)
2018	566	38.5 (7.5)
Diagnosis category		
Schizophrenia/schizoaffective disorder	5466	38.5 (10.4)
Bipolar disorder	1435	37.5 (8.7)

## Discussion

In a large cohort of cases with first diagnosis of SMI drawn from EHRs over a 10-year period, we sought to investigate the level of recording of HbA1c over this time period in a large mental health records database. To our knowledge this is the first such investigation, although attention to dysglycaemia in SMI is an increasing priority in mental healthcare.

A particular feature of this study was that we sought to enhance available data in structured fields with what we believe to be a novel algorithm derived using NLP to identify additional values recorded in text. The level of HbA1c initially available in the analysed sample from structured data fields alone (ie, that imported automatically from laboratory reports) was 19.1%. This rose to 32.2% after the NLP algorithm was applied, representing a clinically important 68% increase, backed up by good performance of the algorithm itself in terms of precision and recall results when compared with a manually annotated gold standard. This indicates wider provision and knowledge of HbA1c data (eg, from broader laboratory networks and presumably via general practitioner communications) than would be readily available for monitoring purposes at a single provider. It therefore underlines the importance of considering text fields in mental healthcare for sources of data, potentially developing and evaluating algorithms for more widespread multisite use. We also evaluated supplementing data further by looking at whether patients had any glucose level recorded, but this produced only marginal further data and was not considered further.

Considering the availability of HbA1c data in the analysed sample, we found that HbA1c recording has increased over time, although was still only present in 42% of those with SMI at its peak in 2016. Although the overall mean interval from SMI diagnosis to first HbA1c recording was relatively short (2 years), data availability will clearly accumulate over time, hence the slightly lower recording levels in 2018 cases closer to the end of the inclusion period.

Our results suggest that recording rates of HbA1c in mental healthcare remain below 50%, despite electronic enhancements such as automatic transfer of local laboratory results and major quality improvement initiatives such as The Commissioning for Quality and Innovation.^5^ Similar findings have been reported from other cohorts, although we were not able to find any study designs directly comparable to our own. For example, Canadian individuals with diabetes and schizophrenia were found to have lower levels of recommended testing than those with diabetes but without schizophrenia;^9^ the former group in that study also had higher rates of diabetes-related hospital visits than the latter, and there is clearly a need to clarify whether lower recorded monitoring results in higher observed adverse consequences. The level of HbA1c recording in people with schizophrenia in that study was 36% over a 2-year period, although the findings are not directly comparable because all of the Canadian cohort had diagnosed diabetes, whereas ours was an unselected sample. An Australian study also reported on diabetes and prediabetes prevalence (14% and 19%, respectively) in psychiatric inpatients who had had no HbA1c recorded in the preceding 3 months;^10^ this again was not directly comparable, although does highlight a general need to screen more regularly.

HbA1c recording has become a focus for UK mental health services in recent years, as a result of government targets being put in place. HbA1c has also gradually taken over from blood glucose as a metric for monitoring or screening for diabetes, although fasting blood glucose levels are preferred after newly starting medication or changes in medication, so that idiosyncratic changes in glucose control can be identified. Although there has been an increase in the years following the introduction of The Commissioning for Quality and Innovation (CQUIN) initiative in 2014,^11^ there are still a lot of patients without a record of this important test, and it is important to understand why. One possibility is that levels are checked in primary care but are not transcribed into the mental healthcare record. This is something we anticipate investigating further in future, taking advantage of CRIS linkages with primary care records for a proportion of the mental health service’s catchment area;^12 13^ however, the lack of recording in the mental health record remains problematic given the level of care required in that sector, the importance of diabetes as a comorbidity in SMI, and the concern about weight gain and impaired glucose tolerance as an adverse effect of some psychotropic medications. Alternatively, as suggested by the Canadian study cited earlier, it is also possible that people with SMI may receive suboptimal screening and monitoring of dysglycaemia across both primary and specialist care sectors, which is also clearly a cause for concern.

We sought to investigate demographic and clinical factors associated with the presence of recorded HbA1c, which we believe is a novel initiative, as we were not able to identify comparable previous research. Associations with younger age may reflect a higher perceived need for screening shortly after onset, and latterly new incentivisation (CQUIN) initiatives around screening were focused particularly on early intervention services; however, it is clearly concerning that recorded levels are less common in older patients who will be at higher risk of dysglycaemia. Higher prevalence of recorded values in men, in non-white ethnic groups and in patients with schizophreniform compared with bipolar disorder diagnoses, on the other hand, may reflect a recognition of higher risk status in these groups.

The mean recorded HbA1c level was 38.3 mmol/mol which is within the normal range, and the SD of 12.3 indicates a sizeable overlap in the distribution of levels with those that would be considered as dysglycaemic (≥48 mmol/mol), consistent with the recognised higher risk in SMI.^14^ In a US cross-sectional study of 114 patients with schizophrenia or schizoaffective disorder aged 26–65 years the mean level of HbA1c was reported as 6.1% which approximates as 43.2 mmol/mol.^15^ This is higher than our estimate, although may be accounted for by the relatively high mean age (48.3 years) of that cohort. In studies of patients presenting with first diagnosis psychosis, lower mean HbA1c levels of 5% (approximating 31.2 mmol/mol) have been described^16 17^ which may reflect the earlier stage of illness captured. In another review of first-diagnosis psychosis, there were relatively few studies identified providing data on HbA1c^18^ which makes this study even more key in understanding how HbA1c is being captured within a mental health setting.

Strengths of the study included the large naturalistic samples, the long time period under investigation and the focus on first diagnosis of SMI, which is a key time for intervention. Considering generalisability, although data are derived from one service provider (SLAM), numerous component teams provide the data and there may therefore be within-provider differences in HbA1c recording, which might be more sizeable than between-provider differences; however, it should be borne in mind that SLAM’s catchment contains a highly socially and culturally mixed population and urban and semiurban settings, resulting in a particular focus on care for SMI, so there is a need for further evaluation and data across broader services and catchments. Considering HbA1c measurement, although the NLP algorithm had a high precision and recall, neither was 100% and therefore there may be findings missed. Given the increase in recording over time, which might have introduced bias from more selective recording in earlier years and preferential monitoring of blood glucose rather than HbA1c in earlier years, we carried out sensitivity analyses restricting to post-2010 results, although these did not alter primary findings substantially. As described above, we were only able to investigate HbA1c recording within mental healthcare and so cannot exclude additional data existing in primary care records; furthermore, we could not identify any other study quantifying the level of primary care HbA1c recording in people with SMI. HbA1c assessment in primary care unknown to secondary care is likely to be minimal in the absence of established diabetes; however, empirical data are required to verify this. Information was also limited on potential confounding factors, including comorbidities, other measures of metabolic syndrome and lifestyle factors such as diet, exercise and smoking. Finally, the focus of the study was on patients at the time of their first diagnosis within a specific mental health service, which does not necessarily equate to the timing of the first ever diagnosis.

In conclusion, the overall level of HbA1c recording was low and further research is needed into ways in which both measurement and recording can be more effectively optimised in these clinical populations.

## Supplementary Material

Reviewer comments

Author's manuscript

## Data Availability

Data are available upon reasonable request. On request, and after appropriate arrangements, the data employed in this study can be viewed within the secure system firewall.

## References

[1] Chang C-K, Hayes RD, Perera G, et al. Life expectancy at birth for people with serious mental illness, substance use disorders, and depressive disorders from a secondary mental health care case register in London, UK. PLoS One 2011;6:e19590. 10.1371/journal.pone.0019590 21611123 PMC3097201

[2] Garcia-Rizo C, Kirkpatrick B, Fernandez-Egea E, et al. Abnormal glycemic homeostasis at the onset of serious mental illnesses: a common pathway. Psychoneuroendocrinology 2016;67:70–5. 10.1016/j.psyneuen.2016.02.001 26878465 PMC4844848

[3] Sengupta S, Parrilla-Escobar MA, Klink R, et al. Are metabolic indices different between drug-naïve first-episode psychosis patients and healthy controls? Schizophr Res 2008;102:329–36. 10.1016/j.schres.2008.02.013 18396386

[4] Solmi M, Fiedorowicz J, Poddighe L, et al. Disparities in screening and treatment of cardiovascular diseases in patients with mental disorders across the world: systematic review and meta-analysis of 47 observational studies. Am J Psychiatry 2021;178:793–803. 10.1176/appi.ajp.2021.21010031 34256605

[5] NICE QS80. Quality statement 6: assessing physical health | psychosis and schizophrenia NICE. 2015. Available: https://www.nice.org.uk/guidance/qs80/chapter/quality-statement-6-assessing-physical-health [Accessed Sep 2021].

[6] Solmi M, Firth J, Miola A, et al. Disparities in cancer screening in people with mental illness across the world versus the general population: prevalence and comparative meta-analysis including 4 717 839 people. Lancet Psychiatry 2020;7:52–63. 10.1016/S2215-0366(19)30414-6 31787585

[7] Perera G, Broadbent M, Callard F, et al. Cohort profile of the South London and Maudsley NHS foundation trust biomedical research centre (SLAM BRC) case register: current status and recent enhancement of an electronic mental health record-derived data resource. BMJ Open 2016;6:e008721. 10.1136/bmjopen-2015-008721 PMC4785292 26932138

[8] Maudsley BRC. Available: https://www.maudsleybrc.nihr.ac.uk/facilities/clinical-record-interactive-search-cris/cris-natural-language-processing/Accessed [Accessed Jul 2020].

[9] Kurdyak P, Vigod S, Duchen R, et al. Diabetes quality of care and outcomes: comparison of individuals with and without schizophrenia. Gen Hosp Psychiatry 2017;46:7–13. 10.1016/j.genhosppsych.2017.02.001 28622820

[10] Naidu P, Churilov L, Kong A, et al. Using routine hemoglobin A1c testing to determine the glycemic status in psychiatric inpatients. Front Endocrinol (Lausanne) 2017;8:53. 10.3389/fendo.2017.00053 28396652 PMC5366933

[11] NICE. Cg178 psychosis and schizophrenia in adults: prevention and management; 2014.

[12] Woodhead C, Ashworth M, Broadbent M, et al. Cardiovascular disease treatment among severe mental illness patients: a data linkage study between primary and secondary care. Br J Gen Pract 2016;66:e374–81. 10.3399/bjgp16X685189 27114210 PMC4871302

[13] Woodhead C, Cunningham R, Ashworth M, et al. Cervical and breast cancer screening uptake among women with serious mental illness: a data linkage study. BMC Cancer 2016;16:819. 10.1186/s12885-016-2842-8 27769213 PMC5073417

[14] Taylor D, Young C, Mohamed R, et al. Undiagnosed impaired fasting glucose and diabetes mellitus amongst inpatients receiving antipsychotic drugs. J Psychopharmacol 2005;19:182–6. 10.1177/0269881105049039 15728440

[15] Lee EE, Martin AS, Tu X, et al. Childhood adversity and schizophrenia: the protective role of resilience in mental and physical health and metabolic markers. J Clin Psychiatry 2018;79:17m11776. 10.4088/JCP.17m11776 PMC6464641 29701938

[16] Yang W, Zheng L, Zheng B, et al. A meta-analysis of abnormal glucose metabolism in first-episode drug-naive schizophrenia. Psychiatr Danub 2020;32:46–54. 10.24869/psyd.2020.46 32303029

[17] Pillinger T, Beck K, Gobjila C, et al. Impaired glucose homeostasis in first-episode schizophrenia: a systematic review and meta-analysis. JAMA Psychiatry 2017;74:261–9. 10.1001/jamapsychiatry.2016.3803 28097367 PMC6352957

[18] Perry BI, McIntosh G, Weich S, et al. The association between first-episode psychosis and abnormal glycaemic control: systematic review and meta-analysis. Lancet Psychiatry 2016;3:1049–58. 10.1016/S2215-0366(16)30262-0 27720402

