# Supplementary material for: HbA1c recording in patients following a first diagnosis of serious mental illness: the South London and Maudsley Biomedical Research Centre case register
Source: BMJ Open. 2023 Jul 18;13(7):e069635. doi: 10.1136/bmjopen-2022-069635 (PMC10357777; doi:10.1136/bmjopen-2022-069635)
Supplement: Author's manuscript [file bmjopen-2022-069635.draft_revisions.pdf]

BMJ Open is committed to open peer review. As part of this commitment we make the peer review history of every article we publish publicly available.

When an article is published we post the peer reviewers' comments and the authors' responses online. We also post the versions of the paper that were used during peer review. These are the versions that the peer review comments apply to.

The versions of the paper that follow are the versions that were submitted during the peer review process. They are not the versions of record or the final published versions. They should not be cited or distributed as the published version of this manuscript.

BMJ Open is an open access journal and the full, final, typeset and author-corrected version of record of the manuscript is available on our site with no access controls, subscription charges or pay-per-view fees (http://bmjopen.bmj.com).

If you have any questions on BMJ Open's open peer review process please email info.bmjopen@bmj.com

BMJ Open

HbA1c recording in patients following a first diagnosis of serious mental illness: The South London and Maudsley Biomedical Research Centre Case Register

Journal:	*BMJ Open*
Manuscript ID	bmjopen-2022-069635
Article Type:	Original research
Date Submitted by the Author:	31-Oct-2022

Complete List of Authors:	Bell, Nikeysha; King's College London Institute of Psychiatry Psychology and Neuroscience, psychological medicine<br>Perera, Gayan; Institute of Psychiatry, Psychological Medicine<br>Stubbs, B; King's College London, Institute of Psychiatry, Psychology and Neuroscience, Psychosis Studies; South London and Maudsley NHS Foundation Trust, National Psychosis Service<br>Gaughran, Fiona; Institute of Psychiatry, Psychology and Neuroscience, Psychosis Studies; South London and Maudsley NHS Foundation Trust, National Psychosis Service<br>Stewart, Robert; King's College London, Institute of Psychiatry; South London and Maudsley NHS Foundation Trust, National Psychosis Service

Keywords:	Health informatics < BIOTECHNOLOGY & BIOINFORMATICS, General diabetes < DIABETES & ENDOCRINOLOGY, EPIDEMIOLOGY, Quality in health care < HEALTH SERVICES ADMINISTRATION & MANAGEMENT, MENTAL HEALTH, PSYCHIATRY

SCHOLARONE™
Manuscripts

I, the Submitting Author has the right to grant and does grant on behalf of all authors of the Work (as defined in the below author licence), an exclusive licence and/or a non-exclusive licence for contributions from authors who are: i) UK Crown employees; ii) where BMJ has agreed a CC-BY licence shall apply, and/or iii) in accordance with the terms applicable for US Federal Government officers or employees acting as part of their official duties; on a worldwide, perpetual, irrevocable, royalty-free basis to BMJ Publishing Group Ltd ("BMJ") its licensees and where the relevant Journal is co-owned by BMJ to the co-owners of the Journal, to publish the Work in this journal and any other BMJ products and to exploit all rights, as set out in our licence.

The Submitting Author accepts and understands that any supply made under these terms is made by BMJ to the Submitting Author unless you are acting as an employee on behalf of your employer or a postgraduate student of an affiliated institution which is paying any applicable article publishing charge ("APC") for Open Access articles. Where the Submitting Author wishes to make the Work available on an Open Access basis (and intends to pay the relevant APC), the terms of reuse of such Open Access shall be governed by a Creative Commons licence – details of these licences and which Creative Commons licence will apply to this Work are set out in our licence referred to above.

Other than as permitted in any relevant BMJ Author's Self Archiving Policies, I confirm this Work has not been accepted for publication elsewhere, is not being considered for publication elsewhere and does not duplicate material already published. I confirm all authors consent to publication of this Work and authorise the granting of this licence.

For peer review only

HbA1c recording in patients following a first diagnosis of serious mental illness: The South London and Maudsley Biomedical Research Centre Case Register

Nikeysha Bell,[1] Gayan Perera,[1] Brendon Stubbs,[1,2] Fiona Gaughran,[1,2] Robert Stewart.[1,2]

[1]Kings College London (Institute of Psychiatry, Psychology and Neuroscience), London, United Kingdom

[2]South London and Maudsley NHS Foundation Trust, London, United Kingdom

Corresponding Author: Nikeysha Bell, Institute of Psychiatry, Psychology and Neuroscience, Kings College London, SLAM BRC Nucleus, Mapother House, Maudsley Hospital, De Crespigny Park,London, SE5 8AZ Nikeysha.bell@kcl.ac.uk

For peer review only

Abstract

Objectives: To investigate factors associated with the recording of HbA1c in people with first diagnoses of SMI in a large mental healthcare provider, and factors associated with HbA1c levels, when recorded. To our knowledge this is the first such investigation, although attention to dysglycaemia in SMI is an increasing priority in mental healthcare

Method: Using electronic mental health records data, we ascertained patients with first SMI diagnoses (schizophrenia, schizoaffective disorder, bipolar disorder) from 2008-2018. First-recorded HbA1c levels were ascertained from routine local laboratory data and supplemented by a natural language processing algorithm for extracting recorded values in text fields (precision 0.89%, recall 0.93%). Age, gender, ethnic group, year of diagnosis, and SMI diagnosis were investigated as covariates in relation to recording or not of HbA1c and first recorded levels.

Results: Of 21,462 patients in the sample (6546 bipolar disorder; 14,916 schizophrenia or schizoaffective disorder; mean age 38.8years, 49% female), 4106 (19.1%) had at least one HbA1c result recorded from laboratory data, increasing to 6901 (32.2%) following natural language processing. HbA1c recording was independently more likely in non-white ethnic groups (Black compared to White: odds ratio 2.45, 95% CI 2.29-2.62), and was negatively associated with age (odds ratio per year increase 0.93, 0.92-0.95), female gender (0.83, 0.78-0.88), and bipolar disorder (0.49, 0.45-0.52). In those with HbA1c recorded, higher levels were independently associated with older age, men, non-white ethnic groups, schizophreniform compared to bipolar disorder diagnoses, and more recent years.

Conclusions: Over a ten-year period, relatively low level of recording of HbA1c was observed, although this has increased over time and ascertainment was increased with text extraction. For those in groups with higher HbA1c, there was more likely to be a recorded

For peer review only

HbA1c. It remains important to improve the routine monitoring of dysglycaemia in these at-risk disorders.

Strengths and limitations:

Strengths

- Large naturalistic samples
- Long time period under investigation
- Focus on first diagnosis

Limitations

- Focus on specific catchment area
- Does not account for information within primary care

For peer review only

Background

People with serious mental illness (SMI) have a substantially reduced life expectancy [1] presenting a major public health challenge. Amongst a range of specific health inequalities, type 2 diabetes mellitus (T2DM) incidence rates are higher than expected [2], potentially accounting for at least some of the mortality gap and providing an important opportunity for health improvement initiatives. As well as unfavourable lifestyle factors, antipsychotic medications may play at least some part in the high prevalence of T2DM through accelerating glucose dysregulation [3]. Other factors such as diagnostic overshadowing and under screening and subsequent treatment could also compound this issue and have been demonstrated in people with established SMI with cardiovascular disease[18]. Identifying predictors of emergent glucose dysregulation would allow interventions to be directed towards those most at risk, thus creating potential for more precise approaches to clinical and preventative care to reduce the risk of diabetes. In order to have maximum effectiveness, early screening is essential.

Despite these opportunities, further work is needed to clarify how feasible it is to predict T2DM, identify it earlier, or mitigate against its complications in people affected by SMI. HbA1c is a commonly used measure of dysglycaemia and is expected to be regularly recorded in people receiving mental health care; for example, it is recommended for English mental health services [4]. Investigating HbA1c recording levels in routine electronic health records (EHRs) is in important first step in this process, including whether this is consistent across all levels of treatment and care; however, to our knowledge this has not received previous attention. First diagnosis in people with psychotic disorders has been identified as the optimal time to screen and prevent physical health conditions developing, including T2DM, but limited data is available on screening levels in this critical time period [5].

For peer review only

The aim of this study was to investigate rates of HbA1c recording in the electronic mental health records of people with newly diagnosed SMI (schizophrenia, schizoaffective disorder or bipolar disorder presenting for the first time) receiving care from a large mental healthcare provider. Further, we specifically sought to investigate the factors associated with HbA1c levels being recorded or not (including changes in recording rates over time), to determine the extent to which ascertained HbA1c recording could be increased through a novel natural language processing (NLP) algorithm applied to free text, and to describe factors associated with HbA1c levels when recorded.

For peer review only

Methods

Sample

The analysed sample was assembled using the Clinical Record Interactive Search (CRIS) data resource at the South London and Maudsley NHS Foundation Trust (SLAM). SLAM is one of Europe's largest mental healthcare providers, serving over 1.2 million residents of four south London boroughs (Lambeth, Southwark, Lewisham, Croydon). With services covering all ages and specialties, SLAM has used fully electronic health records for over ten years and its CRIS platform, set up in 2008 [6], allows researcher access to de-identified data from the full record within a robust governance framework. CRIS has received research ethics approval as a data resource for secondary analyses (Oxford Research Ethics Committee C, reference 18/SC/0372). Because large volumes of information in mental health clinical records are primarily recorded in text, a range of constructs are now quantifiable at scale using NLP algorithms developed for CRIS [7].

Using CRIS, we extracted data on all patients with a first diagnosis of contact between 1st January 2008 and 31st December 2018 that resulted in a recorded diagnosis of schizophrenia, schizoaffective disorder, or bipolar disorder. As described in the Results, analysed data were subsequently restricted to those from 2010 onwards because of limited data for earlier years. If the patient had more than one HbA1c result, the result closest to diagnosis date was used.

Measures

Serum HbA1c data were available from two sources: i) pre-structured imported data from local laboratories from which this assay had been requested by SLAM; ii) information recorded in free text fields such as case notes and clinical correspondence. In order to render information available for analysis from the latter, a set of NLP rules were created to identify

For peer review only

HbA1c as a recorded entity and to extract the numeric value attributed to that assay. From a corpus of 250 randomly selected documents, annotation features were built over a training set. The algorithm derived from this training set was then evaluated against manually annotated gold standards. Once the precision (positive predictive value) and recall (sensitivity) were judged to be at a good enough standard in the development phase, the algorithm was run over CRIS as a whole using a cloud-based processing platform and the results compiled and evaluated formally. The developed NLP algorithm achieved a precision of 89% and a recall of 93% against the gold standard in an independent manually annotated evaluation dataset extracted from CRIS. HbA1c levels were harmonised, as required, so that all were expressed in mmol/mol units.

The following covariates were additionally extracted: age, gender, ethnicity, first SMI diagnosis recorded (categorised into schizophreniform [including schizophrenia or schizoaffective disorder] or bipolar), and the year of first recorded diagnosis.

Statistical analysis

The sample was initially described and unadjusted associations between first SMI diagnosis with HbA1c recording or not as a binary outcome, first investigating laboratory data alone and then supplementing this with data from the natural language processing algorithm. Next, a logistic regression analysis was used to investigate independence of these associations, entering all covariates simultaneously. Within those with HbA1c levels recorded, mean levels were compared by covariate groups, followed by a linear regression analysis of HbA1c as a dependent variable to investigate independence. Finally, given low levels of recording in earlier years, a sensitivity analysis was carried out restricting the sample to those diagnosed in 2010 or after.

For peer review only

Patient and Public Involvement

At project planning stage the Service User Advisory Group were shown initial ideas and plans and asked thoughts and suggestions on these ideas. These were taken on board and informed in part some of the research question.

For peer review only

Results

A sample of 21,462 patients was initially characterised based on the study inclusion criteria for the years 2008-18. From the laboratory data, we ascertained 4106 patients (19.1%) with at least one HbA1c result recorded. When we supplemented this with the NLP algorithm, the number with at least one HbA1c result recorded rose to 6901 (32.2%; a 68% increase); however, the further addition of glucose data did not substantially increase recording results further. The mean (SD) interval between the date of diagnosis and that of the first recorded HbA1c result was 2.1 (2.5) years.

Factors associated with the recording of HbA1c are displayed in Table 1. Considering HbA1c derived from laboratory values or NLP, data were most likely in the 20-29-year age group, in men compared to women, and recording was more common in all minority ethnic groups compared to the White group, particularly so for the Black group, and in those with a schizophreniform rather than bipolar disorder diagnosis. Prevalence of recorded HbA1c rose consistently from 2010 apart from the 2018 year of data extraction. In adjusted analyses (Table 2), associations with all covariates remained statistically significant; however, odds ratios were reduced in strength (towards the null) for gender, diagnosis and named ethnic minority groups; they were strengthened for diagnosis year.

In those with HbA1c data, mean levels are displayed by covariate status in Table 3, and independent associations from linear regression models are displayed in Table 4. In summary, higher HbA1c levels were associated with older age, male gender, and earlier years of diagnosis; they were also higher in Black, Asian, and other ethnic groups compared to White groups, in those with missing ethnic group data, and in those with schizophreniform compared to bipolar diagnoses.

For peer review only

Table 1. Sample characteristics and prevalence of data on HbA1c and/or glucose levels

	Number	HbA1c – lab value		HbA1c – lab value or NLP-derived		HbA1c or glucose	
		% present	Chi-square (df), p-value	% present	Chi-square (df), p-value	% present	Chi-square (df), p-value
Total sample	21462	19.1		32.2		35.2	
Age group							
<20	2660	20.0		30.3		35.9	
20-29	5237	26.0	314.9 (4)	37.9	129.9 (4)	40.6	143.36 (4)
30-39	4791	20.1	<0.001	32.4	<0.001	36.0	<0.001
40-49	3712	16.2		30.9		33.2	
50+	5062	12.8		27.8		29.7	
Gender							
Female	10516	17.9	20.5 (1)	30.1	40.8 (1)	33.2	32.5 (1)
Male	10942	20.3	<0.001	34.1	<0.001	36.9	<0.001
Ethnicity							
White	10923	15.0		26.8		30.2	
Black	5138	29.5	467.0 (4)	47.3	663.8 (4)	49.8	584.4 (4)
Asian	1250	22.5	<0.001	36.0	<0.001	38.7	<0.001
Mixed	631	20.3		33.4		36.9	
Other	1748	19.6		32.4		35.2	
Diagnosis year							
2008	1853	11.4		23.0		24.8	
2009	1859	9.8		22.9		24.4	
2010	2067	11.2	730.3 (10)	23.7	458.5 (10)	25.2	588.0 (10)
2011	1985	12.5	<0.001	25.3	<0.001	27.9	<0.001
2012	1961	14.3		27.5		33.0	
2013	2000	18.9		32.3		37.1	
2014	2010	23.4		39.7		43.3	
2015	1990	24.4		38.4		41.1	
2016	2056	29.3		42.0		45.1	
2017	2129	29.7		41.3		43.8	
2018	1552	24.8		36.5		39.7	
Diagnosis							
Schizophrenia / schizoaffective disorder	14916	21.7	213.2 (1)	36.6	452.1 (1)	39.5	414.7 (1)
Bipolar disorder	6546	13.2	<0.001	21.9	<0.001	25.1	<0.001

For peer review only

Table 2. Logistic regression analysis of factors associated with recorded HbA1c presence in the electronic mental health record for years 2008-18

Characteristic	Unadjusted model 2008-2018			Mutually adjusted model 2008-2018			Mutually adjusted model 2010-2018		
	Odds ratio	95% CI		Odds ratio	95% CI		Odds ratio	95% CI	
Age (10y increment)	0.93	0.92	0.95	0.94	0.93	0.96	0.93	0.92	0.95
Female gender	0.83	0.78	0.88	0.91	0.84	0.97	0.87	0.81	0.93
Ethnicity									
White	Ref.			Ref.			Ref.		
Black	2.45	2.29	2.62	2.20	2.04	2.36	2.16	2.00	2.34
Asian	1.54	1.36	1.74	1.43	1.26	1.62	1.52	1.33	1.75
Mixed	1.37	1.16	1.63	1.21	1.02	1.45	1.14	0.94	1.37
Other	1.31	1.17	1.46	1.19	1.06	1.33	1.23	1.10	1.39
Bipolar disorder diagnosis	0.49	0.45	0.52	0.57	0.53	0.61	0.57	0.53	0.62
Diagnosis year (per 2008-2018 increment)	1.10	1.10	1.12	1.13	1.12	1.15	1.14	1.13	1.16

For peer review only

Table 3. Distributions of first recorded HbA1c levels

	Number with at least one HbA1c recording	Mean (SD) HbA1c first recorded	P value
Total sample	6901	38.3 (10.5)	
Age group			<0.001
<20	807	35.8 (6.0)	
20-29	1985	36.2 (7.2)	
30-39	1554	38.1 (9.8)	
40-49	1147	40.2 (10.0)	
50+	1408	43.5 (14.9)	
Gender			<0.001
Female	3162	38.0 (9.1)	
Male	3736	38.6 (10.8)	
Missing		34 .0 (2.8)	
Ethnicity			<0.001
White	2924	37.6 (9.6)	
Black	2428	39.1 (11.2)	
Asian	450	39.5 (10.1)	
Mixed	211	38.1 (9.3)	
Other	566	37.6 (7.7)	
Missing	322	37.5 (7.2)	
Year of referral			<0.001
2008	426	39.8 (11.2)	
2009	426	40.0 (12.6)	
2010	490	39.7 (11.7)	
2011	503	39.5 (13.0)	
2012	539	38.0 (10.4)	
2013	645	38.0 (10.5)	
2014	798	37.2 (9.0)	
2015	765	38.0 (9.8)	
2016	863	38.3 (8.9)	
2017	880	37.5 (9.5)	
2018	566	38.5 (7.5)	
Diagnosis category			<0.001
Schizophrenia/schizoaffective disorder	5466	38.5 (10.4)	
Bipolar disorder	1435	37.5 (8.7)	

For peer review only

Table 4. Linear regression analysis of factors associated with HbA1c levels in the electronic mental health record

Characteristic	Unadjusted model 2008-2018			Mutually adjusted model 2008-2018			Mutually adjusted model 2010-2018		
	B-coefficient	95% CI		B-coefficient	95%CI		B-coefficient	95%CI	
Age (10y increment)	-0.72	-0.84	-0.60	-0.61	-0.73	-0.48	-0.62	-0.76	-0.48
Female gender	-1.09	-1.50	-0.67	-0.69	-1.13	-0.26	-0.80	-1.30	-0.31
Ethnicity									
White	Ref.			Ref.			Ref.		
Black	5.80	5.28	6.32	5.01	4.49	5.53	5.23	4.62	5.83
Asian	3.29	2.37	4.20	2.79	1.90	3.69	3.33	2.30	4.36
Mixed	2.06	0.80	3.31	0.97	-0.28	2.20	0.55	-0.83	1.94
Other	1.67	0.88	2.46	0.80	0.03	1.58	1.08	0.22	1.93
Bipolar disorder diagnosis	-3.46	-3.91	-3.01	-2.33	-2.82	-1.84	-2.57	-3.12	-2.01
Diagnosis year (1y increment)	0.78	0.71	0.84	0.90	0.83	0.97	1.03	0.93	1.12

For peer review only

Discussion

In a large cohort of cases with first diagnosis of SMI drawn from electronic health records over a ten-year period, we sought to investigate the level of recording of HbA1c, and the levels of recorded HbA1c over this time period in a large mental health records database. To our knowledge this is the first such investigation, although attention to dysglycaemia in SMI is an increasing priority in mental healthcare.

A particular feature of this study was that we sought to enhance available data in structured fields with what we believe to be a novel algorithm derived using natural language processing to identify additional values recorded in text. The level of HbA1c initially available in the analysed sample from structured data fields alone (i.e., that imported automatically from laboratory reports) was 19.1%. This rose to 32.2% after the NLP algorithm was applied, representing a clinically important 68% increase, backed up by good performance of the algorithm itself in terms of precision and recall results when compared to a manually annotated gold standard. This indicates wider provision and knowledge of HbA1c data (e.g., from broader laboratory networks and presumably via general practitioner communications) than would be readily available for monitoring purposes at a single provider. It therefore underlines the importance of considering text fields in mental healthcare for sources of data, potentially developing and evaluating algorithms for more widespread multi-site use. We also evaluated supplementing data further by looking at whether patients had any glucose level recorded, but this produced only marginal further data and was not considered further.

For peer review only

Considering the availability of HbA1c data in the analysed sample, we found that HbA1c recording has increased over time, although was still only present in 42% of those with SMI at its peak in 2016. Although the overall mean interval from SMI diagnosis to first HbA1c recording was relatively short (2 years), data availability will clearly accumulate over time, hence the slightly lower recording levels in 2018 cases closer to the end of the inclusion period.

Our results suggest that recording rates of HbA1c in mental healthcare remain below 50%, despite electronic enhancements such as automatic transfer of local laboratory results and major quality improvement initiatives such as The Commissioning for Quality and Innovation [4] Similar findings have been reported from other cohorts, although we were not able to find any study designs directly comparable to our own. For example, Canadian individuals with diabetes and schizophrenia were found to have lower levels of recommended testing than those with diabetes but without schizophrenia [8]; the former group in that study also had higher rates of diabetes-related hospital visits than the latter, and there is clearly a need to clarify whether lower recorded monitoring results in higher observed adverse consequences. The level of HbA1c recording in people with schizophrenia in that study was 36% over a 2-year period, although the findings are not directly comparable because all of the Canadian cohort had diagnosed diabetes, whereas ours was an unselected sample. An Australian study also reported on diabetes and pre-diabetes prevalence (14% and 19% respectively) in psychiatric inpatients who had had no HbA1c recorded in the preceding three months [9]; this again was not directly comparable, although does highlight a general need to screen more regularly.

HbA1c recording has become a focus for UK mental health services in recent years, as a result of government targets being put in place. HbA1c has also gradually taken over from blood glucose as a metric for monitoring or screening for diabetes, although fasting blood

For peer review only

glucose levels are preferred after newly starting medication or changes in medication, so that idiosyncratic changes in glucose control can be identified. Although there has been an increase in the years following the introduction of the CQUIN initiative in 2014 [10], there are still a lot of patients without a record of this important test, and it is important to understand why. One possibility is that levels are checked in primary care but are not transcribed into the mental healthcare record. This is something we anticipate investigating further in future, taking advantage of CRIS linkages with primary care records for a proportion of the mental health service's catchment area [11,12]; however, the lack of recording in the mental health record remains problematic given the level of care required in that sector, the importance of diabetes as a comorbidity in SMI, and the concern about weight gain and impaired glucose tolerance as an adverse effect of some psychotropic medications. Alternatively, as suggested by the Canadian study cited earlier, it is also possible that people with SMI may receive suboptimal screening and monitoring of dysglycaemia across both primary and specialist care sectors, which is also clearly a cause for concern.

We sought to investigate demographic and clinical factors associated with the presence of recorded HbA1c, which we believe is a novel initiative, as we were not able to identify comparable previous research. Associations with younger age may reflect a higher perceived need for screening shortly after onset, and latterly new incentivisation (CQUIN) initiatives around screening were focused particularly on early intervention services; however, it is clearly concerning that recorded levels are less common in older patients who will be at higher risk of dysglycaemia. Higher prevalence of recorded values in men, in non-white ethnic groups, and in patients with schizophreniform compared to bipolar disorder diagnoses, on the other hand, may reflect a recognition of higher risk status in these groups.

The mean recorded HbA1c level was 38.3 mmol/mol which is within the normal range, and the standard deviation of 12.3 indicates a sizeable overlap in the distribution of levels with

For peer review only

those that would be considered as dysglycaemic (>/=48mmol/mol), consistent with the recognised higher risk in SMI [13]. In a US cross-sectional study of 114 patients with schizophrenia or schizoaffective disorder aged 26-65 years the mean level of HbA1c was reported as 6.1% which approximates as 43.2mmol/mol [14]. This is higher than our estimate, although may be accounted for by the relatively high mean age (48.3 years) of that cohort. In studies of patients presenting with first diagnosis psychosis, lower mean HbA1c levels of 5% (approximating 31.2 mmol/mol) have been described [15] which may reflect the earlier stage of illness captured. A review of measures of glucose homeostasis in first-diagnosis schizophrenia found elevations of fasting plasma glucose, plasma insulin, and insulin resistance in patients compared to controls, but no significant differences in HbA1c levels [16];the authors advised caution in interpreting this finding because of small sample sizes, although it was further confirmed in a more recent review [15] suggesting that divergences in this parameter might occur at a later point in the development of the mental disorder – again, a reason for better monitoring. In another review of first-diagnosis psychosis, there were relatively few studies identified providing data on HbA1c [17].

Strengths of the study included the large naturalistic samples, the long time period under investigation and the focus on first diagnosis of SMI, which is a key time for intervention. Considering generalisability, although data are derived from one service provider, component teams are numerous and between-team differences in HbA1c recording are likely to be more sizeable than between-provider differences; however, it should be borne in mind that SLAM's catchment contains a highly socially and culturally mixed population and urban and semi-urban settings, resulting in a particular focus on care for SMI, so there is a need for further evaluation and data across broader services and catchments. Considering HbA1c measurement, although the NLP algorithm had a high precision and recall, neither was 100% and therefore there may be findings missed. Given the increase in recording over time, which

For peer review only

might have introduced bias from more selective recording in earlier years and preferential monitoring of blood glucose rather than HbA1c in earlier years, we carried out sensitivity analyses restricting to post-2010 results, although these did not alter primary findings substantially. As described above, we were only able to investigate HbA1c recording and levels within mental healthcare and so cannot exclude additional data existing in primary care records. Information was also limited on potential confounding factors, including comorbidities, other measures of metabolic syndrome, and lifestyle factors such as diet, exercise, and smoking. Finally, the focus of the study was on patients at the time of their first diagnosis within a specific mental health service, which does not necessarily equate to the timing of the first ever diagnosis.

In conclusion, although recording of HbA1c tended to be more likely in groups at higher risk, based on analysed levels, the overall level of recording was low and further research is needed into ways in which both measurement and recording can be more effectively optimised in these clinical populations.

For peer review only

Funding

NB, FG, GP and RS are part-funded by the National Institute for Health Research (NIHR) Biomedical Research Centre at the South London and Maudsley NHS Foundation Trust and King's College London; FG and RS are part-funded by the National Institute for Health Research (NIHR) Applied Research Collaboration South London (NIHR ARC South London) at King's College Hospital NHS Foundation Trust; RS is part-funded by the DATAMIND HDR UK Mental Health Data Hub (MRC grant MR/W014386). FG is also part-funded by the Maudsley Charity.BS holds an NIHR Advanced fellowship (NIHR301206, 2021-2026). The views expressed are those of the authors and not necessarily those of the NIHR or the Department of Health and Social Care.

Declaration of interests

RS declares research support received in the last 3 years from Janssen, GSK and Takeda, and royalties from Oxford University Press. FG has received honoraria from, Lundbeck, Otsuka and Sunovion. BS is on the Editorial board of Ageing Research Reviews, Mental Health and Physical Activity, The Journal of Evidence Based Medicine, and The Brazilian Journal of Psychiatry. BS has received honorarium from a co-edited a book on exercise and mental illness, advisory work from ASICS Europe BV & FitXR for unrelated work.

Contributions

The study was originally conceived by NB, RS, FG and BS. Data were prepared and analysed by NB and GP. The report was written by NB with input from all authors. All authors critically revised the manuscript and approved the final version.

Ethics approval statement

For peer review only

The CRIS platform is approved as a source of anonymised data for secondary research use by Oxford Research Ethics committee C (Reference 18/SC/0372)

Data sharing statement

Data are available on reasonable request. The CRIS database has received ethical approval for secondary analysis: Oxford REC C, reference 18/SC/0372. On request, and after appropriate arrangements, the data employed in this study can be viewed within the secure system firewall.

For peer review only

References

1. Chang C-K, Hayes RD, Perera G, *et al.*Life expectancy at birth for people with serious mental illness, substance use disorders, and depressive disorders from a secondary mental health care case register in London, UK. *PLoS One* 2011; 6(5): e19590.

2. Garcia-Rizo C, Kirkpatrick B, Fernandez-Egea E,*et al.* Abnormal glycemic homeostasis at the onset of serious mental illnesses: a common pathway. *Psychoneuroendocrinology* 2016; 67: 70-75.

3. Sengupta S, Parrilla-Escobar M, Klink R, *et al*. Are metabolic indices different between drug-naïve first-episode psychosis patients and healthy controls? *Schizophrenia Research* 2008; 102: 329-336.

4. NICE QS80 ,2015 Quality statement 6: Assessing physical health | Psychosis and schizophrenia NICE ttps://www.nice.org.uk/guidance/qs80/chapter/quality-statement-6-assessing-physical-health Accessed September 2021

5. Solmi M, Firth J,Miola A *et al*. Disparities in cancer screening in people with mental illness across the world versus the general population: prevalence and comparative meta-analysis including 4 717 839 people.*Lancet Psychiatry*. 2020 Jan;7(1):52-63

6. Perera G, Broadbent M, Callard F, *et al*. Cohort profile of the South London and Maudsley NHS Foundation trust biomedical research centre (SLAM BRC) case register: current status and recent enhancement of an electronic mental health Record-derived data resource. *BMJ Open* 2016;6:e008721.

7. Maudsley BRC https://www.maudsleybrc.nihr.ac.uk/facilities/clinical-record-interactive-search-cris/cris-natural-language-processing/Accessed July 2020

For peer review only

8. Kurdyak P, Vigod S, Duchen R *et al.* Diabetes quality of care and outcomes: Comparison of individuals with and without schizophrenia, *General Hospital Psychiatry*, 2017 46, 7-13,

9. Naidu P, Churilov L, Kong A, *et al*. Using Routine Hemoglobin A1c Testing to Determine the Glycemic Status in Psychiatric Inpatients. *Front Endocrinol (Lausanne).* 2017;8:53.

10. NICE 2014 CG178 Psychosis and schizophrenia in adults: prevention and management, Accessed December 2020.

11. Woodhead C, Ashworth M, Broadbent, M *et al* Cardiovascular disease treatment among severe mental illness patients: a data linkage study between primary and secondary care. *British Journal of General Practice* 2016a; 66: e374-e381.

12. Woodhead C, Cunningham R, Ashworth, M *et al* Cervical and breast cancer screening uptake among women with serious mental illness: a data linkage study. *BMC Cancer* 2016b; 16: 819.

13. Taylor D, Young C, Mohamed R *et al*. Undiagnosed impaired fasting glucose and diabetes mellitus amongst inpatients receiving antipsychotic drugs. *Journal of Psychopharmacology.* 2005;19(2):182–186

14. Lee, Ellen E, Martin AS, Tu X *et al*. "Childhood Adversity and Schizophrenia: The Protective Role of Resilience in Mental and Physical Health and Metabolic Markers." *The Journal of clinical psychiatry*, 2018;*79*(3), 17m11776.

15. Yang W, Zheng L, Zheng B, *et al*. A Meta-Analysis of Abnormal Glucose Metabolism in First-Episode Drug-Naive Schizophrenia. *Psychiatria Danubia.* 2020;32(1):46-54.

For peer review only

16. Pillinger, Toby Beck K , Gobijila C.*et al.* “Impaired Glucose Homeostasis in First-Episode Schizophrenia: A Systematic Review and Meta-analysis.” *JAMA psychiatry* 2017; 74,3 261-269. doi:10.1001/jamapsychiatry.2016.3803

17. Perry BI, McIntosh G, Weich, S.*et al.* The association between first-episode psychosis and abnormal glycaemic control: systematic review and meta-analysis. *Lancet Psychiatry*. 2016;3(11):1049-1058.

18. Solmi M, Fiedorowicz J, Poddighe L, et al. Disparities in Screening and Treatment of Cardiovascular Diseases in Patients With Mental Disorders Across the World: Systematic Review and Meta-Analysis of 47 Observational Studies. *Am J Psychiatry*. 2021;178(9):793-803.

For peer review only

For peer review only

STROBE Statement—Checklist of items that should be included in reports of ***cohort studies***

	Item No	Recommendation	Page No
**Title and abstract**	1	(*a*) Indicate the study's design with a commonly used term in the title or the abstract<br>(*b*) Provide in the abstract an informative and balanced summary of what was done and what was found	1,2
**Introduction**			
Background/rationale	2	Explain the scientific background and rationale for the investigation being reported	4
Objectives	3	State specific objectives, including any prespecified hypotheses	5
**Methods**			
Study design	4	Present key elements of study design early in the paper	6,7
Setting	5	Describe the setting, locations, and relevant dates, including periods of recruitment, exposure, follow-up, and data collection	6,7
Participants	6	(*a*) Give the eligibility criteria, and the sources and methods of selection of participants. Describe methods of follow-up<br>(*b*) For matched studies, give matching criteria and number of exposed and unexposed	6,7
Variables	7	Clearly define all outcomes, exposures, predictors, potential confounders, and effect modifiers. Give diagnostic criteria, if applicable	6,7
Data sources/ measurement	8*	For each variable of interest, give sources of data and details of methods of assessment (measurement). Describe comparability of assessment methods if there is more than one group	6,7
Bias	9	Describe any efforts to address potential sources of bias	6,7
Study size	10	Explain how the study size was arrived at	6,7
Quantitative variables	11	Explain how quantitative variables were handled in the analyses. If applicable, describe which groupings were chosen and why	
Statistical methods	12	(*a*) Describe all statistical methods, including those used to control for confounding<br>(*b*) Describe any methods used to examine subgroups and interactions<br>(*c*) Explain how missing data were addressed<br>(*d*) If applicable, explain how loss to follow-up was addressed<br>(*e*) Describe any sensitivity analyses	7
**Results**			
Participants	13*	(a) Report numbers of individuals at each stage of study—eg numbers potentially eligible, examined for eligibility, confirmed eligible, included in the study, completing follow-up, and analysed<br>(b) Give reasons for non-participation at each stage<br>(c) Consider use of a flow diagram	9
Descriptive data	14*	(a) Give characteristics of study participants (eg demographic, clinical, social) and information on exposures and potential confounders<br>(b) Indicate number of participants with missing data for each variable of interest<br>(c) Summarise follow-up time (eg, average and total amount)	9
Outcome data	15*	Report numbers of outcome events or summary measures over time	9

For peer review only - http://bmjopen.bmj.com/site/about/guidelines.xhtml

Main results	16	(*a*) Give unadjusted estimates and, if applicable, confounder-adjusted estimates and their precision (eg, 95% confidence interval). Make clear which confounders were adjusted for and why they were included<br>(*b*) Report category boundaries when continuous variables were categorized<br>(*c*) If relevant, consider translating estimates of relative risk into absolute risk for a meaningful time period	9-13
Other analyses	17	Report other analyses done—eg analyses of subgroups and interactions, and sensitivity analyses	9-13
**Discussion**			
Key results	18	Summarise key results with reference to study objectives	14-18
Limitations	19	Discuss limitations of the study, taking into account sources of potential bias or imprecision. Discuss both direction and magnitude of any potential bias	17,18
Interpretation	20	Give a cautious overall interpretation of results considering objectives, limitations, multiplicity of analyses, results from similar studies, and other relevant evidence	17,18
Generalisability	21	Discuss the generalisability (external validity) of the study results	17,18
**Other information**			
Funding	22	Give the source of funding and the role of the funders for the present study and, if applicable, for the original study on which the present article is based	19

*Give information separately for exposed and unexposed groups.

Note: An Explanation and Elaboration article discusses each checklist item and gives methodological background and published examples of transparent reporting. The STROBE checklist is best used in conjunction with this article (freely available on the Web sites of PLoS Medicine at http://www.plosmedicine.org/, Annals of Internal Medicine at http://www.annals.org/, and Epidemiology at http://www.epidem.com/). Information on the STROBE Initiative is available at http://www.strobe-statement.org.

For peer review only

BMJ Open

HbA1c recording in patients following a first diagnosis of serious mental illness: The South London and Maudsley Biomedical Research Centre Case Register

Journal:	*BMJ Open*
Manuscript ID	bmjopen-2022-069635.R1
Article Type:	Original research
Date Submitted by the Author:	10-May-2023
Complete List of Authors:	Bell, Nikeysha; King's College London Institute of Psychiatry Psychology and Neuroscience, psychological medicine<br>Perera, Gayan; Institute of Psychiatry, Psychological Medicine<br>Chandran, David; King's College London, Institute of Psychiatry<br>Stubbs, B; King's College London, Institute of Psychiatry, Psychology and Neuroscience, Psychosis Studies; South London and Maudsley NHS Foundation Trust, National Psychosis Service<br>Gaughran, Fiona; Institute of Psychiatry, Psychology and Neuroscience, Psychosis Studies; South London and Maudsley NHS Foundation Trust, National Psychosis Service<br>Stewart, Robert; King's College London, Institute of Psychiatry; South London and Maudsley NHS Foundation Trust, National Psychosis Service
**Primary Subject Heading**:	Mental health
Secondary Subject Heading:	Health informatics, Diabetes and endocrinology, Mental health
Keywords:	Health informatics < BIOTECHNOLOGY & BIOINFORMATICS, General diabetes < DIABETES & ENDOCRINOLOGY, EPIDEMIOLOGY, Quality in health care < HEALTH SERVICES ADMINISTRATION & MANAGEMENT, MENTAL HEALTH, PSYCHIATRY

SCHOLARONE™
Manuscripts

I, the Submitting Author has the right to grant and does grant on behalf of all authors of the Work (as defined in the below author licence), an exclusive licence and/or a non-exclusive licence for contributions from authors who are: i) UK Crown employees; ii) where BMJ has agreed a CC-BY licence shall apply, and/or iii) in accordance with the terms applicable for US Federal Government officers or employees acting as part of their official duties; on a worldwide, perpetual, irrevocable, royalty-free basis to BMJ Publishing Group Ltd ("BMJ") its licensees and where the relevant Journal is co-owned by BMJ to the co-owners of the Journal, to publish the Work in this journal and any other BMJ products and to exploit all rights, as set out in our licence.

The Submitting Author accepts and understands that any supply made under these terms is made by BMJ to the Submitting Author unless you are acting as an employee on behalf of your employer or a postgraduate student of an affiliated institution which is paying any applicable article publishing charge ("APC") for Open Access articles. Where the Submitting Author wishes to make the Work available on an Open Access basis (and intends to pay the relevant APC), the terms of reuse of such Open Access shall be governed by a Creative Commons licence – details of these licences and which Creative Commons licence will apply to this Work are set out in our licence referred to above.

Other than as permitted in any relevant BMJ Author's Self Archiving Policies, I confirm this Work has not been accepted for publication elsewhere, is not being considered for publication elsewhere and does not duplicate material already published. I confirm all authors consent to publication of this Work and authorise the granting of this licence.

For peer review only

HbA1c recording in patients following a first diagnosis of serious mental illness: The South London and Maudsley Biomedical Research Centre Case Register

Nikeysha Bell,[1] Gayan Perera,[1] David Chandran,[1] Brendon Stubbs,[1,2] Fiona Gaughran,[1,2] Robert Stewart.[1,2]

[1]Kings College London (Institute of Psychiatry, Psychology and Neuroscience), London, United Kingdom

[2]South London and Maudsley NHS Foundation Trust, London, United Kingdom

Corresponding Author: Nikeysha Bell, Institute of Psychiatry, Psychology and Neuroscience, Kings College London, SLAM BRC Nucleus, Mapother House, Maudsley Hospital, De Crespigny Park,London, SE5 8AZ Nikeysha.bell@kcl.ac.uk

For peer review only

Abstract

Objectives: To investigate factors associated with the recording of HbA1c in people with first diagnoses of SMI in a large mental healthcare provider, and factors associated with HbA1c levels, when recorded. To our knowledge this is the first such investigation, although attention to dysglycaemia in SMI is an increasing priority in mental healthcare.

Design: The study was primarily descriptive in nature, seeking to ascertain the frequency of HbA1c recording in the mental healthcare sector for people following first SMI diagnosis.

Settings: A large mental health care provider, the South London and Maudsley (SLAM) NHS Trust.

Participants: Using electronic mental health records data, we ascertained patients with first SMI diagnoses (schizophrenia, schizoaffective disorder, bipolar disorder) from 2008-2018.

Outcome measures: Recording or not of HbA1c level was ascertained from routine local laboratory data and supplemented by a natural language processing algorithm for extracting recorded values in text fields (precision 0.89%, recall 0.93%). Age, gender, ethnic group, year of diagnosis, and SMI diagnosis were investigated as covariates in relation to recording or not of HbA1c and first recorded levels.

Results: Of 21,462 patients in the sample (6546 bipolar disorder; 14,916 schizophrenia or schizoaffective disorder; mean age 38.8years, 49% female), 4106 (19.1%) had at least one HbA1c result recorded from laboratory data, increasing to 6901 (32.2%) following natural language processing. HbA1c recording was independently more likely in non-white ethnic groups (Black compared to White: odds ratio 2.45, 95% CI 2.29-2.62), and was negatively associated with age (odds ratio per year increase 0.93, 0.92-0.95), female gender (0.83, 0.78-0.88), and bipolar disorder (0.49, 0.45-0.52).

For peer review only

Conclusions: Over a ten-year period, relatively low level of recording of HbA1c was observed, although this has increased over time and ascertainment was increased with text extraction. It remains important to improve the routine monitoring of dysglycaemia in these at-risk disorders.

Strengths and limitations:

Strengths

- First study of this kind, we believe
- Seeking to understand whether HbA1c is being recorded/sufficiently recorded in the SMI population (a current NHS target)
- High precision and recall for the NLP algorithm (precision 0.89%, recall 0.93%).

Limitations

- Focus on specific catchment area
- Does not account for information within primary care

For peer review only

Background

People with serious mental illness (SMI) have a substantially reduced life expectancy [1] presenting a major public health challenge. Amongst a range of specific health inequalities, type 2 diabetes mellitus (T2DM) incidence rates are higher than expected [2], potentially accounting for at least some of the mortality gap and providing an important opportunity for health improvement initiatives. As well as unfavourable lifestyle factors, antipsychotic medications may play at least some part in the high prevalence of T2DM through accelerating glucose dysregulation [3]. Other factors such as diagnostic overshadowing and under screening and subsequent treatment could also compound this issue and have been demonstrated in people with established SMI with cardiovascular disease [4]. Identifying predictors of emergent glucose dysregulation would allow interventions to be directed towards those most at risk, thus creating potential for more precise approaches to clinical and preventative care to reduce the risk of diabetes. In order to have maximum effectiveness, early screening is essential.

Despite these opportunities, further work is needed to clarify how feasible it is to predict T2DM, identify it earlier, or mitigate against its complications in people affected by SMI. HbA1c is a commonly used measure of dysglycaemia and is expected to be regularly recorded in people receiving mental health care; for example, it is recommended for English mental health services [5]. Investigating HbA1c recording levels in routine electronic health records (EHRs) is in important first step in this process, including whether this is consistent across all levels of treatment and care; however, to our knowledge this has not received previous attention. First diagnosis in people with psychotic disorders has been identified as the optimal time to screen and prevent physical health conditions developing, including T2DM, but limited data is available on screening levels in this critical time period [6].

For peer review only

The aim of this study was to investigate rates of HbA1c recording in the electronic mental health records of people with newly diagnosed SMI (schizophrenia, schizoaffective disorder or bipolar disorder presenting for the first time) receiving care from a large mental healthcare provider. Further, we specifically sought to investigate the factors associated with HbA1c levels being recorded or not (including changes in recording rates over time), to determine the extent to which ascertained HbA1c recording could be increased through a novel natural language processing (NLP) algorithm applied to free text, and to describe distributions of HbA1c levels when recorded.

For peer review only

Methods

Sample

The analysed sample was assembled using the Clinical Record Interactive Search (CRIS) data resource at the South London and Maudsley NHS Foundation Trust (SLAM). SLAM is one of Europe's largest mental healthcare providers, serving over 1.2 million residents of four south London boroughs (Lambeth, Southwark, Lewisham, Croydon). With services covering all ages and specialties, SLAM has used fully electronic health records for over ten years and its CRIS platform, set up in 2008 [7], allows researcher access to de-identified data from the full record within a robust governance framework. CRIS has received research ethics approval as a data resource for secondary analyses (Oxford Research Ethics Committee C, reference 18/SC/0372). Because large volumes of information in mental health clinical records are primarily recorded in text, a range of constructs are now quantifiable at scale using natural language processing (NLP) algorithms developed for CRIS [8].

Using CRIS, we extracted data on all patients with a first diagnosis of contact between 1st January 2008 and 31st December 2018 that resulted in a recorded diagnosis of schizophrenia, schizoaffective disorder, or bipolar disorder. As described in the Results, analysed data were subsequently restricted to those from 2010 onwards because of limited data for earlier years. If the patient had more than one HbA1c result, the result closest to diagnosis date was used.

Measures

Serum HbA1c data were available from two sources: i) pre-structured imported data from local laboratories from which this assay had been requested by SLAM; ii) information recorded in free text fields such as case notes and clinical correspondence. In order to render information available for analysis from the latter, a rules-based NLP algorithm was created to

For peer review only

identify HbA1c as a recorded entity and to extract the numeric value attributed to that assay. From a corpus of 250 randomly selected documents, annotation features were first built over a training set. The algorithm rules sought to identify instances of HbA1c recording and the associated score where this was being recorded in relation to the patient (i.e., rather than anyone else). Any units (% or mmol/mol) were allowable and any number of decimal places (if any). Primary exclusions were HbA1c mentions without a score – for example, text simply indicating that levels were within the normal range or that a measurement had taken place (i.e., without a value described). The algorithm derived from this training set was then evaluated against manually annotated gold standards. Once the precision (positive predictive value) and recall (sensitivity) were judged to be at a good enough standard in the development phase, the algorithm was run over CRIS as a whole using a cloud-based processing platform and the results compiled and evaluated formally. The developed NLP algorithm achieved a precision of 89% and a recall of 93% against the gold standard in an independent manually annotated evaluation dataset extracted from CRIS.

The following covariates were additionally extracted: age, gender, ethnicity, first SMI diagnosis recorded (categorised into schizophreniform [including schizophrenia or schizoaffective disorder] or bipolar), and the year of first recorded diagnosis. Recorded glucose level was further evaluated as a measure of dysglycaemia.

Statistical analysis

The sample was initially described and unadjusted associations between first SMI diagnosis with HbA1c recording or not as a binary outcome, first investigating laboratory data alone and then supplementing this with data from the natural language processing algorithm. Next, a logistic regression analysis was used to investigate independence of these associations,

For peer review only

entering all covariates simultaneously. Finally, given low levels of recording in earlier years, a sensitivity analysis was carried out restricting the sample to those diagnosed in 2010 or after.

Patient and Public Involvement

At project planning stage, members of a Service User Advisory Group were shown initial ideas and plans and asked for thoughts and suggestions relating to a broader body of work (a PhD programme for NB) of which this study was a component. The group consisted of services users (of SLAM services) with lived experience of mental health problems. They were very keen on finding out the results of the HbA1c recording and encouraged data linkage to primary care (which was subsequently carried out for a nested cohort of people with pre-existing diabetes, to be published separately). Suggestions were taken on board and informed, in part, some of the research questions.

For peer review only

Results

A sample of 21,462 patients was initially characterised based on the study inclusion criteria for the years 2008-18. From the laboratory data, we ascertained 4106 patients (19.1%) with at least one HbA1c result recorded. When we supplemented this with the NLP algorithm, the number with at least one HbA1c result recorded rose to 6901 (32.2%; a 68% increase); however, the further addition of glucose data did not substantially increase recording results further (Table 1). The mean (SD) interval between the date of diagnosis and that of the first recorded HbA1c result was 2.1 (2.5) years.

Factors associated with the recording of HbA1c are displayed in Table 1. Considering HbA1c derived from laboratory values or NLP, data were most likely in the 20-29-year age group, in men compared to women, and recording was more common in all minority ethnic groups compared to the White group, particularly so for the Black group, and in those with a schizophreniform rather than bipolar disorder diagnosis. Prevalence of recorded HbA1c rose consistently from 2010 apart from the 2018 year of data extraction. In adjusted analyses (Table 2), associations with all covariates remained statistically significant; however, odds ratios were reduced in strength (towards the null) for gender, diagnosis and named ethnic minority groups; they were strengthened for diagnosis year. In those with HbA1c data, mean levels are displayed by covariate status in Table 3.

For peer review only

Table 1. Sample characteristics and prevalence of data on HbA1c and/or glucose levels

	Number	HbA1c – lab value		HbA1c – lab value or NLP-derived		HbA1c or glucose	
		% present	Chi-square (df), p-value	% present	Chi-square (df), p-value	% present	Chi-square (df), p-value
Total sample	21462	19.1		32.2		35.2	
Age group							
<20	2660	20.0		30.3		35.9	
20-29	5237	26.0	314.9 (4)	37.9	129.9 (4)	40.6	143.36 (4)
30-39	4791	20.1	<0.001	32.4	<0.001	36.0	<0.001
40-49	3712	16.2		30.9		33.2	
50+	5062	12.8		27.8		29.7	
Gender							
Female	10516	17.9	20.5 (1)	30.1	40.8 (1)	33.2	32.5 (1)
Male	10942	20.3	<0.001	34.1	<0.001	36.9	<0.001
Ethnicity							
White	10923	15.0		26.8		30.2	
Black	5138	29.5	467.0 (4)	47.3	663.8 (4)	49.8	584.4 (4)
Asian	1250	22.5	<0.001	36.0	<0.001	38.7	<0.001
Mixed	631	20.3		33.4		36.9	
Other	1748	19.6		32.4		35.2	
Diagnosis year							
2008	1853	11.4		23.0		24.8	
2009	1859	9.8		22.9		24.4	
2010	2067	11.2	730.3 (10)	23.7	458.5 (10)	25.2	588.0 (10)
2011	1985	12.5	<0.001	25.3	<0.001	27.9	<0.001
2012	1961	14.3		27.5		33.0	
2013	2000	18.9		32.3		37.1	
2014	2010	23.4		39.7		43.3	
2015	1990	24.4		38.4		41.1	
2016	2056	29.3		42.0		45.1	
2017	2129	29.7		41.3		43.8	
2018	1552	24.8		36.5		39.7	
Diagnosis							
Schizophrenia / schizoaffective disorder	14916	21.7	213.2 (1)	36.6	452.1 (1)	39.5	414.7 (1)
Bipolar disorder	6546	13.2	<0.001	21.9	<0.001	25.1	<0.001

For peer review only

Table 2. Logistic regression analysis of factors associated with recorded HbA1c presence in the electronic mental health record for years 2008-18

Characteristic	Unadjusted model 2008-2018	Mutually adjusted model 2008-2018	Mutually adjusted model 2010-2018
	Odds ratio (95% CI)	Odds ratio (95% CI)	Odds ratio (95% CI)
Age (10y increment)	0.93 (0.92,0.95)	0.94 (0.93,0.96)	0.93 (0.92,0.95)
Female gender	0.83 (0.78,0.88)	0.91 (0.84,0.97)	0.87 (0.81,0.93)
Ethnicity			
White	Ref.	Ref.	Ref.
Black	2.45 (2.29,2.62)	2.2 (2.04,2.36)	2.16 (2.00,2.34)
Asian	1.54 (1.36,1.74)	1.43 (1.26,1.62)	1.52 (1.33,1.75)
Mixed	1.37 (1.16,1.63)	1.21 (1.02,1.45)	1.14 (0.94,1.37)
Other	1.31 (1.17,1.46)	1.19 (1.06,1.33)	1.23 1.10,1.39)
Bipolar disorder diagnosis	0.49 (0.45,0.52)	0.57 (0.53,0.61)	0.57 (0.53,0.62)
Diagnosis year (per 2008-2018 increment)	1.1 (1.10,1.12)	1.13 (1.12,1.15)	1.14 (1.13,1.16)

For peer review only

Table 3. Distributions of first recorded HbA1c levels

	Number with at least one HbA1c recording	Mean (SD) HbA1c first recorded
Total sample	6901	38.3 (10.5)
Age group		
<20	807	35.8 (6.0)
20-29	1985	36.2 (7.2)
30-39	1554	38.1 (9.8)
40-49	1147	40.2 (10.0)
50+	1408	43.5 (14.9)
Gender		
Female	3162	38.0 (9.1)
Male	3736	38.6 (10.8)
Missing		34 .0 (2.8)
Ethnicity		
White	2924	37.6 (9.6)
Black	2428	39.1 (11.2)
Asian	450	39.5 (10.1)
Mixed	211	38.1 (9.3)
Other	566	37.6 (7.7)
Missing	322	37.5 (7.2)
Year of referral		
2008	426	39.8 (11.2)
2009	426	40.0 (12.6)
2010	490	39.7 (11.7)
2011	503	39.5 (13.0)
2012	539	38.0 (10.4)
2013	645	38.0 (10.5)
2014	798	37.2 (9.0)
2015	765	38.0 (9.8)
2016	863	38.3 (8.9)
2017	880	37.5 (9.5)
2018	566	38.5 (7.5)
Diagnosis category		
Schizophrenia/schizoaffective disorder	5466	38.5 (10.4)
Bipolar disorder	1435	37.5 (8.7)

For peer review only

Discussion

In a large cohort of cases with first diagnosis of SMI drawn from electronic health records over a ten-year period, we sought to investigate the level of recording of HbA1c over this time period in a large mental health records database. To our knowledge this is the first such investigation, although attention to dysglycaemia in SMI is an increasing priority in mental healthcare.

A particular feature of this study was that we sought to enhance available data in structured fields with what we believe to be a novel algorithm derived using natural language processing to identify additional values recorded in text. The level of HbA1c initially available in the analysed sample from structured data fields alone (i.e., that imported automatically from laboratory reports) was 19.1%. This rose to 32.2% after the NLP algorithm was applied, representing a clinically important 68% increase, backed up by good performance of the algorithm itself in terms of precision and recall results when compared to a manually annotated gold standard. This indicates wider provision and knowledge of HbA1c data (e.g., from broader laboratory networks and presumably via general practitioner communications) than would be readily available for monitoring purposes at a single provider. It therefore underlines the importance of considering text fields in mental healthcare for sources of data, potentially developing and evaluating algorithms for more widespread multi-site use. We also evaluated supplementing data further by looking at whether patients had any glucose level recorded, but this produced only marginal further data and was not considered further.

Considering the availability of HbA1c data in the analysed sample, we found that HbA1c recording has increased over time, although was still only present in 42% of those with SMI at its peak in 2016. Although the overall mean interval from SMI diagnosis to first HbA1c recording was relatively short (2 years), data availability will clearly accumulate over time,

For peer review only

hence the slightly lower recording levels in 2018 cases closer to the end of the inclusion period.

Our results suggest that recording rates of HbA1c in mental healthcare remain below 50%, despite electronic enhancements such as automatic transfer of local laboratory results and major quality improvement initiatives such as The Commissioning for Quality and Innovation [5] Similar findings have been reported from other cohorts, although we were not able to find any study designs directly comparable to our own. For example, Canadian individuals with diabetes and schizophrenia were found to have lower levels of recommended testing than those with diabetes but without schizophrenia [9]; the former group in that study also had higher rates of diabetes-related hospital visits than the latter, and there is clearly a need to clarify whether lower recorded monitoring results in higher observed adverse consequences. The level of HbA1c recording in people with schizophrenia in that study was 36% over a 2-year period, although the findings are not directly comparable because all of the Canadian cohort had diagnosed diabetes, whereas ours was an unselected sample. An Australian study also reported on diabetes and pre-diabetes prevalence (14% and 19% respectively) in psychiatric inpatients who had had no HbA1c recorded in the preceding three months [10]; this again was not directly comparable, although does highlight a general need to screen more regularly.

HbA1c recording has become a focus for UK mental health services in recent years, as a result of government targets being put in place. HbA1c has also gradually taken over from blood glucose as a metric for monitoring or screening for diabetes, although fasting blood glucose levels are preferred after newly starting medication or changes in medication, so that idiosyncratic changes in glucose control can be identified. Although there has been an increase in the years following the introduction of the CQUIN initiative in 2014 [11], there are still a lot of patients without a record of this important test, and it is important to

For peer review only

understand why. One possibility is that levels are checked in primary care but are not transcribed into the mental healthcare record. This is something we anticipate investigating further in future, taking advantage of CRIS linkages with primary care records for a proportion of the mental health service's catchment area [12,13]; however, the lack of recording in the mental health record remains problematic given the level of care required in that sector, the importance of diabetes as a comorbidity in SMI, and the concern about weight gain and impaired glucose tolerance as an adverse effect of some psychotropic medications. Alternatively, as suggested by the Canadian study cited earlier, it is also possible that people with SMI may receive suboptimal screening and monitoring of dysglycaemia across both primary and specialist care sectors, which is also clearly a cause for concern.

We sought to investigate demographic and clinical factors associated with the presence of recorded HbA1c, which we believe is a novel initiative, as we were not able to identify comparable previous research. Associations with younger age may reflect a higher perceived need for screening shortly after onset, and latterly new incentivisation (CQUIN) initiatives around screening were focused particularly on early intervention services; however, it is clearly concerning that recorded levels are less common in older patients who will be at higher risk of dysglycaemia. Higher prevalence of recorded values in men, in non-white ethnic groups, and in patients with schizophreniform compared to bipolar disorder diagnoses, on the other hand, may reflect a recognition of higher risk status in these groups.

The mean recorded HbA1c level was 38.3 mmol/mol which is within the normal range, and the standard deviation of 12.3 indicates a sizeable overlap in the distribution of levels with those that would be considered as dysglycaemic (>/=48mmol/mol), consistent with the recognised higher risk in SMI [14]. In a US cross-sectional study of 114 patients with schizophrenia or schizoaffective disorder aged 26-65 years the mean level of HbA1c was reported as 6.1% which approximates as 43.2mmol/mol [15]. This is higher than our

For peer review only

estimate, although may be accounted for by the relatively high mean age (48.3 years) of that cohort. In studies of patients presenting with first diagnosis psychosis, lower mean HbA1c levels of 5% (approximating 31.2 mmol/mol) have been described [16,17] which may reflect the earlier stage of illness captured. In another review of first-diagnosis psychosis, there were relatively few studies identified providing data on HbA1c [18] which makes this study even more key in understanding how HbA1c is being captured within a mental health setting.

Strengths of the study included the large naturalistic samples, the long time period under investigation and the focus on first diagnosis of SMI, which is a key time for intervention. Considering generalisability, although data are derived from one service provider (SLAM), numerous component teams provide the data and there may therefore be within-provider differences in HbA1c recording, which might be more sizeable than between-provider differences; however, it should be borne in mind that SLAM's catchment contains a highly socially and culturally mixed population and urban and semi-urban settings, resulting in a particular focus on care for SMI, so there is a need for further evaluation and data across broader services and catchments. Considering HbA1c measurement, although the NLP algorithm had a high precision and recall, neither was 100% and therefore there may be findings missed. Given the increase in recording over time, which might have introduced bias from more selective recording in earlier years and preferential monitoring of blood glucose rather than HbA1c in earlier years, we carried out sensitivity analyses restricting to post-2010 results, although these did not alter primary findings substantially. As described above, we were only able to investigate HbA1c recording within mental healthcare and so cannot exclude additional data existing in primary care records; furthermore, we could not identify any other study quantifying the level of primary care HbA1c recording in people with SMI. HbA1c assessment in primary care unknown to secondary care is likely to be minimal in the absence of established diabetes; however, empirical data are required to verify this.

For peer review only

Information was also limited on potential confounding factors, including comorbidities, other measures of metabolic syndrome, and lifestyle factors such as diet, exercise, and smoking. Finally, the focus of the study was on patients at the time of their first diagnosis within a specific mental health service, which does not necessarily equate to the timing of the first ever diagnosis.

In conclusion, the overall level of HbA1c recording was low and further research is needed into ways in which both measurement and recording can be more effectively optimised in these clinical populations.

For peer review only

Funding

NB, FG, GP and RS are part-funded by the National Institute for Health Research (NIHR) Biomedical Research Centre at the South London and Maudsley NHS Foundation Trust and King's College London; FG and RS are part-funded by the National Institute for Health Research (NIHR) Applied Research Collaboration South London (NIHR ARC South London) at King's College Hospital NHS Foundation Trust; RS is part-funded by the DATAMIND HDR UK Mental Health Data Hub (MRC grant MR/W014386). FG is also part-funded by the Maudsley Charity.BS holds an NIHR Advanced fellowship (NIHR301206, 2021-2026). The views expressed are those of the authors and not necessarily those of the NIHR or the Department of Health and Social Care.

Declaration of interests

RS declares research support received in the last 3 years from Janssen, GSK and Takeda, and royalties from Oxford University Press. FG has received honoraria from, Lundbeck, Otsuka and Sunovion. BS is on the Editorial board of Ageing Research Reviews, Mental Health and Physical Activity, The Journal of Evidence Based Medicine, and The Brazilian Journal of Psychiatry. BS has received honorarium from a co-edited a book on exercise and mental illness, advisory work from ASICS Europe BV & FitXR for unrelated work.

Contributions

The study was originally conceived by NB, RS, FG and BS. Data were prepared and analysed by NB,GP and DC. The report was written by NB with input from all authors. All authors critically revised the manuscript and approved the final version.

Ethics approval statement

For peer review only

The CRIS platform is approved as a source of anonymised data for secondary research use by Oxford Research Ethics Committee C (Reference 18/SC/0372)

Data sharing statement

Data are available on reasonable request. The CRIS database has received ethical approval for secondary analysis: Oxford REC C, reference 18/SC/0372. On request, and after appropriate arrangements, the data employed in this study can be viewed within the secure system firewall.

For peer review only

References

1. Chang C-K, Hayes RD, Perera G, *et al.*Life expectancy at birth for people with serious mental illness, substance use disorders, and depressive disorders from a secondary mental health care case register in London, UK. *PLoS One* 2011; 6(5): e19590.

2. Garcia-Rizo C, Kirkpatrick B, Fernandez-Egea E,*et al.* Abnormal glycemic homeostasis at the onset of serious mental illnesses: a common pathway. *Psychoneuroendocrinology* 2016; 67: 70-75.

3. Sengupta S, Parrilla-Escobar M, Klink R, *et al*. Are metabolic indices different between drug-naïve first-episode psychosis patients and healthy controls? *Schizophrenia Research* 2008; 102: 329-336.

4. Solmi M, Fiedorowicz J, Poddighe L, et al. Disparities in Screening and Treatment of Cardiovascular Diseases in Patients With Mental Disorders Across the World: Systematic Review and Meta-Analysis of 47 Observational Studies. *Am J Psychiatry*. 2021;178(9):793-803.

5. NICE QS80 ,2015 Quality statement 6: Assessing physical health | Psychosis and schizophrenia NICE ttps://www.nice.org.uk/guidance/qs80/chapter/quality-statement-6-assessing-physical-health Accessed September 2021

6. Solmi M, Firth J,Miola A *et al*. Disparities in cancer screening in people with mental illness across the world versus the general population: prevalence and comparative meta-analysis including 4 717 839 people.*Lancet Psychiatry*. 2020 Jan;7(1):52-63

7. Perera G, Broadbent M, Callard F, *et al*. Cohort profile of the South London and Maudsley NHS Foundation trust biomedical research centre (SLAM BRC) case

For peer review only

register: current status and recent enhancement of an electronic mental health Record-derived data resource. *BMJ Open* 2016;6:e008721.

8. Maudsley BRC https://www.maudsleybrc.nihr.ac.uk/facilities/clinical-record-interactive-search-cris/cris-natural-language-processing/Accessed July 2020

9. Kurdyak P, Vigod S, Duchen R *et al.* Diabetes quality of care and outcomes: Comparison of individuals with and without schizophrenia, *General Hospital Psychiatry*, 2017 46, 7-13,

10. Naidu P, Churilov L, Kong A, *et al*. Using Routine Hemoglobin A1c Testing to Determine the Glycemic Status in Psychiatric Inpatients. *Front Endocrinol (Lausanne).* 2017;8:53.

11. NICE 2014 CG178 Psychosis and schizophrenia in adults: prevention and management, Accessed December 2020.

12. Woodhead C, Ashworth M, Broadbent, M *et al* Cardiovascular disease treatment among severe mental illness patients: a data linkage study between primary and secondary care. *British Journal of General Practice* 2016a; 66: e374-e381.

13. Woodhead C, Cunningham R, Ashworth, M *et al* Cervical and breast cancer screening uptake among women with serious mental illness: a data linkage study. *BMC Cancer* 2016b; 16: 819.

14. Taylor D, Young C, Mohamed R *et al*. Undiagnosed impaired fasting glucose and diabetes mellitus amongst inpatients receiving antipsychotic drugs. *Journal of Psychopharmacology.* 2005;19(2):182–186

15. Lee, Ellen E, Martin AS, Tu X *et al*. "Childhood Adversity and Schizophrenia: The Protective Role of Resilience in Mental and Physical Health and Metabolic Markers." *The Journal of clinical psychiatry*, 2018;*79*(3), 17m11776.

For peer review only

16. Yang W, Zheng L, Zheng B, *et al*. A Meta-Analysis of Abnormal Glucose Metabolism in First-Episode Drug-Naive Schizophrenia. *Psychiatria Danubia.* 2020;32(1):46-54.

17. Pillinger, Toby Beck K , Gobijila C.*et al.* "Impaired Glucose Homeostasis in First-Episode Schizophrenia: A Systematic Review and Meta-analysis." *JAMA psychiatry* 2017; 74,3 261-269. doi:10.1001/jamapsychiatry.2016.3803

18. Perry BI, McIntosh G, Weich, S.*et al.* The association between first-episode psychosis and abnormal glycaemic control: systematic review and meta-analysis. *Lancet Psychiatry*. 2016;3(11):1049-1058.

For peer review only

For peer review only

For peer review only

STROBE Statement—Checklist of items that should be included in reports of ***cohort studies***

	Item No	Recommendation	Page No
**Title and abstract**	1	(*a*) Indicate the study's design with a commonly used term in the title or the abstract<br>(*b*) Provide in the abstract an informative and balanced summary of what was done and what was found	1,2
**Introduction**			
Background/rationale	2	Explain the scientific background and rationale for the investigation being reported	4
Objectives	3	State specific objectives, including any prespecified hypotheses	5
**Methods**			
Study design	4	Present key elements of study design early in the paper	6,7
Setting	5	Describe the setting, locations, and relevant dates, including periods of recruitment, exposure, follow-up, and data collection	6,7
Participants	6	(*a*) Give the eligibility criteria, and the sources and methods of selection of participants. Describe methods of follow-up<br>(*b*) For matched studies, give matching criteria and number of exposed and unexposed	6,7
Variables	7	Clearly define all outcomes, exposures, predictors, potential confounders, and effect modifiers. Give diagnostic criteria, if applicable	6,7
Data sources/ measurement	8*	For each variable of interest, give sources of data and details of methods of assessment (measurement). Describe comparability of assessment methods if there is more than one group	6,7
Bias	9	Describe any efforts to address potential sources of bias	6,7
Study size	10	Explain how the study size was arrived at	6,7
Quantitative variables	11	Explain how quantitative variables were handled in the analyses. If applicable, describe which groupings were chosen and why	
Statistical methods	12	(*a*) Describe all statistical methods, including those used to control for confounding<br>(*b*) Describe any methods used to examine subgroups and interactions<br>(*c*) Explain how missing data were addressed<br>(*d*) If applicable, explain how loss to follow-up was addressed<br>(*e*) Describe any sensitivity analyses	7
**Results**			
Participants	13*	(a) Report numbers of individuals at each stage of study—eg numbers potentially eligible, examined for eligibility, confirmed eligible, included in the study, completing follow-up, and analysed<br>(b) Give reasons for non-participation at each stage<br>(c) Consider use of a flow diagram	9
Descriptive data	14*	(a) Give characteristics of study participants (eg demographic, clinical, social) and information on exposures and potential confounders<br>(b) Indicate number of participants with missing data for each variable of interest<br>(c) Summarise follow-up time (eg, average and total amount)	9
Outcome data	15*	Report numbers of outcome events or summary measures over time	9

For peer review only

For peer review only - http://bmjopen.bmj.com/site/about/guidelines.xhtml

Main results	16	(*a*) Give unadjusted estimates and, if applicable, confounder-adjusted estimates and their precision (eg, 95% confidence interval). Make clear which confounders were adjusted for and why they were included<br>(*b*) Report category boundaries when continuous variables were categorized<br>(*c*) If relevant, consider translating estimates of relative risk into absolute risk for a meaningful time period	9-13
Other analyses	17	Report other analyses done—eg analyses of subgroups and interactions, and sensitivity analyses	9-13
**Discussion**			
Key results	18	Summarise key results with reference to study objectives	14-18
Limitations	19	Discuss limitations of the study, taking into account sources of potential bias or imprecision. Discuss both direction and magnitude of any potential bias	17,18
Interpretation	20	Give a cautious overall interpretation of results considering objectives, limitations, multiplicity of analyses, results from similar studies, and other relevant evidence	17,18
Generalisability	21	Discuss the generalisability (external validity) of the study results	17,18
**Other information**			
Funding	22	Give the source of funding and the role of the funders for the present study and, if applicable, for the original study on which the present article is based	19

*Give information separately for exposed and unexposed groups.

Note: An Explanation and Elaboration article discusses each checklist item and gives methodological background and published examples of transparent reporting. The STROBE checklist is best used in conjunction with this article (freely available on the Web sites of PLoS Medicine at http://www.plosmedicine.org/, Annals of Internal Medicine at http://www.annals.org/, and Epidemiology at http://www.epidem.com/). Information on the STROBE Initiative is available at http://www.strobe-statement.org.

For peer review only

For peer review only - http://bmjopen.bmj.com/site/about/guidelines.xhtml
